# Magnesium Sulfate Attenuates Lethality and Oxidative Damage Induced by Different Models of Hypoxia in Mice

**DOI:** 10.1155/2020/2624734

**Published:** 2020-12-18

**Authors:** Hamidreza Mohammadi, Amir Shamshirian, Shafagh Eslami, Danial Shamshirian, Mohammad Ali Ebrahimzadeh

**Affiliations:** ^1^Department of Medicinal Chemistry, School of Pharmacy and Pharmaceutical Sciences Research Center, Hemoglobinopathy Institute, Mazandaran University of Medical Sciences, Sari, Iran; ^2^Department of Medical Laboratory Sciences, Student Research Committee, School of Allied Medical Science, Mazandaran University of Medical Sciences, Sari, Iran; ^3^Gastrointestinal Cancer Research Center, Non-Communicable Diseases Institute, Mazandaran University of Medical Sciences, Sari, Iran; ^4^Chronic Respiratory Diseases Research Center, National Research Institute of Tuberculosis and Lung Diseases (NRITLD), Shahid Beheshti University of Medical Sciences, Tehran, Iran

## Abstract

Mg^2+^ is an important cation in our body. It is an essential cofactor for many enzymes. Despite many works, nothing is known about the protective effects of MgSO_4_ against hypoxia-induced lethality and oxidative damage in brain mitochondria. In this study, antihypoxic and antioxidative activities of MgSO_4_ were evaluated by three experimental models of induced hypoxia (asphyctic, haemic, and circulatory) in mice. Mitochondria protective effects of MgSO_4_ were evaluated in mouse brain after induction of different models of hypoxia. Antihypoxic activity was especially pronounced in asphyctic hypoxia, where MgSO_4_ at dose 600 mg/kg showed the same activity as phenytoin, which used as a positive control (*P* < 0.001). In the haemic model, MgSO_4_ at all used doses significantly prolonged latency of death. In circulatory hypoxia, MgSO_4_ (600 mg/kg) doubles the survival time. MgSO_4_ significantly decreased lipid peroxidation and protein carbonyl and improved mitochondrial function and glutathione content in brain mitochondria compared to the control groups. The results obtained in this study showed that MgSO_4_ administration has protective effects against lethality induced by different models of hypoxia and improves brain mitochondria oxidative damage.

## 1. Introduction

The cation Mg^2+^ has an important role in the intracellular process regulation. It is necessary for ATP activity containing calcium current across and within cell membranes for tissues. Moreover, Mg^2+^ exists in more than 300 enzymatic systems through antioxidative properties and is the fourth most abundant cation in our body [1, 2]. Mg^2+^ is an essential cofactor for the activity of kinases and has effects on oxidative phosphorylation, DNA synthesis, lipid hydrolysis, and rates of glycolysis [3, 4]. Subsequently, Mg^2+^ is compulsory in all relating ATP reactions, and variations in this ion may regulate oxidative phosphorylation and substrate in the myocardium [5]. Cardiovascular disease has been associated with diminished Mg^2+^ levels, and a pivotal relationship may be present between mortality from nonocclusive ischemic heart disease and decreased cardiac Mg^2+^ [5, 6]. Myocardial damage consequence of oxidative stress in the heart cells can occur in several conduction problems such as hypoxia. There is evidence that hypoxia reduces cellular ATP, depletes mitochondrial energy, elevates ADP/ATP ratio mainly in the heart muscle cells, and induces oxidative stress [7]. The increased generation of ROS can cause a decrease in the rate of ATP synthesis in mitochondria because of the loss of cytochrome oxidase (Cox) activity. Cox is a chain in the mitochondrial respiratory process, which generates ATP by oxidative phosphorylation. The inhibition of Cox appears to be the main reason for the ATP depletion. A reduced capacity of Cox will cause an increase in ROS production and decrease ATP synthesis [5, 6, 8].

The imbalance between the supply of oxygen and its demand determines organ hypoxia. It occurs mainly in ischemia and heart diseases and leads to numerous deleterious effects and finally resulting in death [9]. Hypoxia causes oxidative stress involving the production of reactive oxygen species [6, 10]. It has been confirmed that the compounds with antioxidant activity can scavenge ROS and are able to show the antihypoxic property [11].

MgSO_4_ is a well-known cation that is vital for cell viability. To the best of our knowledge, there is no report about the protective effects of this ion against lethality and mitochondria oxidative brain damage induced by hypoxia. The present work is aimed at determining the antihypoxic activities and neuroprotective effects of MgSO_4_ to understand a possible mechanism of its action in hypoxia-induced lethality and brain mitochondria oxidative damage protection induced by different models of hypoxia.

## 2. Material and Methods

### 2.1. Chemicals

All chemical agents were prepared from Sigma-Aldrich Company and were used in this study.

### 2.2. Animals

Male Swiss albino mice (21 ± 3 g) were housed in polypropylene cages at 25 ± 1°C and 45-55% relative humidity, with a 12 h light : 12 h dark cycle (lights on at 7 a.m.). The animals had free access to standard pellet and water ad libitum. Experiments were conducted between 8:00 and 15:00 h. All the experimental procedures were performed following the NIH guidelines of the Laboratory Animal Care and Use. The Institutional Animal Ethical Committee of Mazandaran University of Medical Sciences also approved the experimental protocol (with ethics number IR.MAZUMS.REC.93.1001).

### 2.3. Animal Treatment

All of the 208 male mice were randomly divided into 26 groups (eight in each group). Thirteen groups were used for hypoxia-induced methods, and 13 groups were used for the evaluation of brain mitochondria oxidative stress biomarkers after induction of different hypoxia methods.

### 2.4. Asphyctic Hypoxia

The animals were subjected to hypoxia by putting them individually in a tightly closed 300 ml glass container, which was placed underwater in an aquarium of 25°C. The animals had convulsions and died from hypoxia. The latencies for death were recorded. The animals died approximately 1.5-2 min following convulsions. Mice received single i.p. injections of 300, 400, and 500 mg/kg doses of MgSO_4_ or phenytoin (50 mg/kg) as 30 min before they were subjected to hypoxia. The control group was treated with normal saline [12].

### 2.5. Haemic Hypoxia

Forty mice were divided into five groups, each containing eight mice. Mice were injected with sodium nitrite (NaNO_2_) in dose 360 mg/kg i.p. thirty minutes after the i.p. administration of 400, 500, or 600 mg/kg doses of MgSO_4_. The survival time (in min) for each animal was defined as time, measured from the hypoxia induction, caused by the introduction of hypoxia until death. The control group was treated with normal saline.

### 2.6. Circulatory Hypoxia

Forty mice were divided into five groups, each containing eight mice. The groups were treated with normal saline. Thirty minutes after *i.p.* administration of 400, 500, and 600 mg/kg doses of MgSO_4_, NaF (150 mg/kg) was applied *i.p.* to mice, and antihypoxic activity was estimated in minutes as the latent time of evidence of hypoxia [13].

### 2.7. Preparation of the Brain Mitochondria

All animals were sacrificed by cervical decapitation after seven minutes (in haemic and circulatory models) and 25 minutes in the asphyctic model based on our above results. Then, brain tissues were homogenized, and mitochondria were extracted from mouse brains using differential centrifugation. Tris buffer (0.05 M Tris-HCl, 20 mm KCl, 0.25 M sucrose, 2.0 mM MgCl_2_, and 1.0 mM Na_2_HPO_4_, pH = 7.4) was used for suspending of the final mitochondrial pellets. For ROS production assessment, mitochondria were suspended in respiration buffer (0.32 mM sucrose, 20 mM Mops, 10 mM Tris, 0.5 mM MgCl_2_, 50 *μ*M EGTA, 0.1 mM KH_2_PO_4_, and 5 mM sodium succinate). All procedures were done at 4°C [14].

### 2.8. Protein Concentration

The Coomassie blue protein binding method [15] was used for the measurement of mitochondria protein content in the samples.

### 2.9. Lipid Peroxidation (LPO) Measurement

MDA malondialdehyde content was measured by the Zhang et al. method [16]. Briefly, phosphoric acid (0.25 ml, 0.05 M) was added to mitochondrial fractions (0.2 ml, 0.5 mg protein/ml), and then, thiobarbituric acid (TBA) (0.3 ml, 0.2%) was added to them. Afterward, the microtubes were located (30 min) in a boiling water bath. In the end, the samples were placed in an ice bath, and n-butanol (0.4 ml) was added to each sample. Finally, samples were centrifuged at 3500 × g for 10 min, and the MDA formed in the supernatant was determined at 532 nm with an ELISA reader (Tecan, Rainbow Thermo, Austria). Tetramethoxypropane as standard was used in this test.

### 2.10. Protein Carbonyl (PC) Determination

Protein carbonyl content in brain mitochondria was assessed by the spectrophotometric method. Briefly, 500 *μ*l of 20% (*w*/*v*) trichloroacetic acid (TCA) was added to the samples (0.5 mg mitochondrial protein/ml). Afterward, the samples were stored at 4°C for 15 min. The precipitates of the samples were treated with 500 *μ*l of 0.2% DNPH (2,4-dinitrophenylhydrazine) and incubated for 1 hour at room temperature. 55 *μ*l of 100% TCA was used for precipitation of the proteins. Samples were centrifuged and washed with 1000 *μ*l of the ethanol-ethyl acetate (1 : 1, *v*/*v*) mixture. Guanidine hydrochloride (200 *μ*l, 6 M) was added to the samples, and the content of the carbonyl created was calculated by reading the absorbance at 365 nm wavelength [14].

### 2.11. Determination of GSH Content

The content of the GSH in the isolated mitochondria was measured using DTNB by the spectrophotometer method. The developed yellow color by DTNB was recorded at 412 nm. GSH content in each sample was expressed as *μ*M [17].

### 2.12. Assessment of Mitochondrial Toxicity

Mitochondrial toxicity was assessed as an evaluation of mitochondria function in samples by measuring the reduction of MTT (3-[4,5-dimethylthiazol-2-yl]-2,5-diphenyltetrazolium bromide) with minor modification of Ghazi-Khansari et al. [18].

### 2.13. Statistical Analysis

Data were presented as mean ± SD. Analysis of variance (ANOVA) was performed. Newman-Keuls multiple comparison tests were used to determine the differences in means. All *P* values less than 0.05 were regarded as significant.

## 3. Results

**Figure 1:**
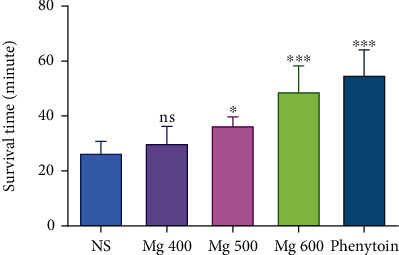
Antihypoxic activities of different doses of MgSO_4_ in the asphyctic hypoxia method in mice. Data are expressed as mean ± SD (*n* = 8).

The results of the asphyctic hypoxia are shown in Figure 1. The effect was dose dependent. MgSO_4_ at doses of 500 and 600 mg/kg significantly (*P* < 0.05 and *P* < 0.001) prolonged the latency for death concerning the control group. At dose 600 mg/kg, MgSO_4_ showed the same activity as phenytoin, which was used as a positive control (*P* > 0.001). Even at the lowest tested dose, 400 mg/kg, it has prolonged survival time (30.20 ± 5.27 min), but this prolongation was not statistically significant from the control (*P* > 0.05).

**Figure 2:**
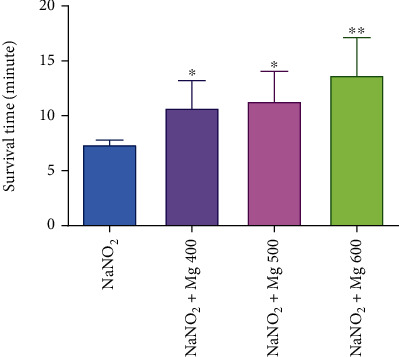
Antihypoxic activities of MgSO_4_ at different doses in induced haemic hypoxia in mice. Data are expressed as mean ± SD (*n* = 8); ^∗^*P* < 0.05 and ^∗∗^*P* < 0.01 compared to control (NaNO_2_).

MgSO_4_ showed good activity in the haemic model (Figure 2). The control group died by induced haemic hypoxia at 7.40 ± 0.46 min. MgSO_4_, at all tested doses, showed statistically significant activity concerning the control. This effect was dose dependent. At the highest measured dose, 600 mg/kg, it has prolonged latency for death to 13.72 ± 2.88 min, which was statistically (*P* < 0.01) significant from the control.

**Figure 3:**
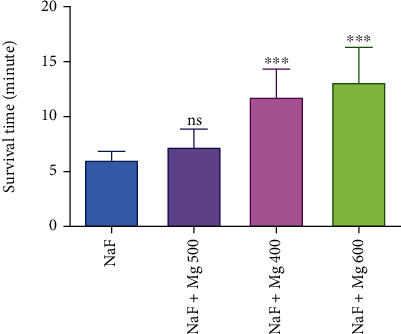
Antihypoxic activities of different doses of MgSO_4_ in circulatory-induced hypoxia in mice. Data are expressed as mean ± SD (*n* = 8); ns: not significant; ^∗∗∗^*P* < 0.001 compared to control (NaF).

The results of the circulatory hypoxia are shown in Figure 3. MgSO_4_ at doses of 500 and 600 mg/kg showed the most potent effect. It significantly prolonged the latency for death concerning the control group (>13.17 ± 2.06*vs*. 6.3 ± 0.74 min, *P* < 0.001). At these doses, it showed doubled survival time, and the effects were dose dependent. At the lowest tested dose (400 mg/kg), it also kept mice alive for 7.28 ± 1.40 min, but this effect was not statistically significant compared to the control (*P* > 0.05).

MDA as a marker of lipid peroxidation was assayed in the brain mitochondria of mice following different methods of induced hypoxia. As shown in Table 1, the amount of MDA formation in mitochondria was decreased significantly (*P* < 0.05) by MgSO_4_ (500 and 600 mg/kg) in haemic induced hypoxia and by MgSO_4_ (600 mg/kg) in hypoxia induced by asphyctic and circulatory methods when compared with the control group of each.

Due to previous findings on the beneficial effects of magnesium in mitochondrial function, we decided to assess the probable benefit role of this metal on the mitochondrial antioxidant status. Mitochondrial GSH was measured in isolated brain mitochondria after induced hypoxia with different methods. Levels of glutathione contents were significantly (*P* < 0.05) increased in the phenytoin and MgSO_4_ (500 mg/kg) groups compared to control in asphyctic condition, and it was increased in the MgSO_4_ (500 mg/kg) group in haemic (NaNO_2_) hypoxia condition compared to control (NS+NaNO_2_) group (Table 1). Protein carbonyl as a marker of protein oxidation in mitochondria was measured in this study. Compared to each control group (NS, NS+NaF, and NS+NaNO_2_), PC significantly increased by treatment with phenytoin, MgSO_4_ (400, 500, and 600 mg/kg) in asphyctic, MgSO_4_ (400 and 500 mg/kg) in circulatory, and MgSO_4_ (400 mg/kg) in haemic hypoxia groups (Table 1).

**Figure 4:**
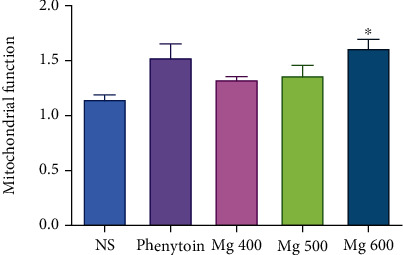
Effect of different doses of MgSO_4_ and phenytoin on mitochondrial function after induced hypoxia by the asphyctic method. Values represented as mean ± SD (*n* = 8). ^∗^*P* < 0.05 compared with the control group (NS).

**Figure 5:**
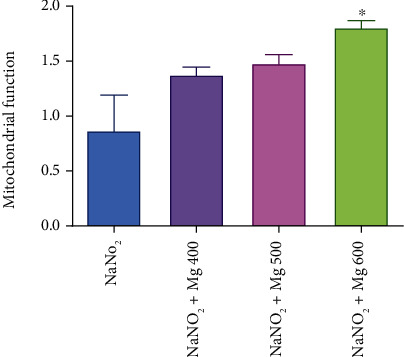
Effect of different doses of MgSO_4_ on mitochondrial function after induced hypoxia by the haemic (NaNO_2_) method. Data are presented as mean ± SD (*n* = 8). ^∗^*P* < 0.05 compared with the control group (NaNO_2_).

**Figure 6:**
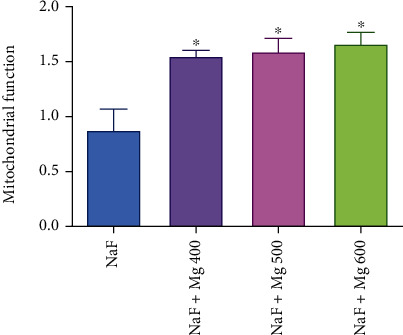
Effect of different doses of MgSO_4_ on mitochondrial function after induced hypoxia by the circulatory (NaF) method. Data are presented as mean ± SD (*n* = 8). ^∗^*P* < 0.05 compared with the control group (NaF).

As shown in Figures 4 and 5, MTT reduction to Formosan, as a mitochondrial toxicity assessment, significantly (*P* < 0.05) increases by MgSO_4_ (600 mg/kg) in the hypoxia induced via asphyctic and haemic methods (44% and 66%, respectively). Mitochondria function was increased significantly (*P* < 0.05) by all doses of MgSO_4_ (23%, 33%, and 67%) in circulatory hypoxia when compared to the control group (Figure 6).

## 4. Discussion

This study was undertaken to evaluate how probably the administration of MgSO_4_ would reduce oxidative damage in hypoxia induced with different methods. The results illustrated that MgSO_4_ improved oxidative stress induced by different hypoxia in a proper manner.

Hypoxia produces intense physiologic stress and induces a wide range of lethal effects at the cellular level. The brain with consumption of a large amount of oxygen is very vulnerable to the low levels of oxygen [11] because it has a high content of polyunsaturated fatty acids, which easily undergo oxidation [19]. The animals' survival time in a sealed container reflects the antihypoxic activity directly.

The lack of oxygen in the brain leads to inevitable changes in the structure and function of cerebral tissue. Thus, any medication that allows the brain to fight the consequences of ischemia or hypoxia would be a great therapeutic agent [19]. Throughout the past decades, a variety of different experimental models have been developed that could be used for testing antihypoxic and anti-ischemic drug effects *in vivo*.

Free radicals make the role of signaling species in different standard physiological procedures, but excessive production of such radicals leads to biological material damage. The elevated levels of ROS in hypoxia are the result of the accumulation of reducing equivalents in the mitochondrial electron transport system [20]. The ROS effects can be mainly evident in specific tissues such as the brain because it uses about one-fifth of the basal oxygen [21]. Given the efforts in developing treatments to reduce the oxidative stress effects, evidence indicates the protecting role of antioxidants in a variety of diseases [22].

In this study, statistically significant antihypoxic activities were established in some doses of MgSO_4_ in all experimental models of induced hypoxia in mice. The effects were dose dependent. Nearly at all tested doses, MgSO_4_ statistically showed significant activity with respect to the controls. A relationship between oxidative metabolism and cholinergic function has been reported during the investigations of NaNO_2_ on brain metabolism [23].

Chemical hypoxia induced by the injection of NaNO_2_ will reduce the blood oxygen capacity by changing hemoglobin to methemoglobin. The deadly dose is injected 30 min after the MgSO_4_ therapy. Immediately after injection, the animals are positioned in small cages, and the time between the NaNO_2_ injection and respiration cessation is recorded. Magnesium sulfate indicated an excellent activity in the haemic model (Figure 2).

There are literature data that the administration of NaF, which induces circulatory hypoxia, increases the blood histamine content and decreases the oxygen-carrying capacity. MgSO_4_ at 600 mg/kg showed the highest activity. The mechanism of induced protective action might be due to the MgSO_4_ antioxidant activity. Because there is no standard drug for haemic and circulatory hypoxic models, the results of this study were compared to those of control groups.

Mg is the most abundant cation with antioxidative properties that exist in more than 300 enzymatic systems. Mg is necessary for ATP activity and is vital in different processes, such as current the calcium across and within the cell membranes in tissues [1, 2].

Tissue hypoxia can induce cell death by inhibition of the activity of complexes I to IV in the mitochondria respiratory chain. Moreover, the reduction of mitochondrial transmembrane potential and depletion of intracellular ATP contents can occur, which finally elevates the ADP/ATP ratio in the cell [24, 25]. The consequence of these processes lactate dehydrogenase releases and damaging the cell membrane integrity can occur. It was demonstrated that hypoxia induces acidosis and depletion of mitochondrial energy in cells. Induced oxidative damage leads to the release of proteolytic enzymes and DNA fragmentations, processes which lead to cell death [26, 27]. It was reported that Mg has a beneficial role in the prevention of cell death by inhibiting calcium accumulation and improving cell metabolism [5]. The present results indicated significant improvement in oxidative biomarkers when MgSO_4_ was applied to induced hypoxic mice. The mechanism of Mg as an antioxidant defence system is not so precise and is still a matter of debate, but the beneficial function of Mg in oxidative stress and damage to molecules and cells has been proven by several studies [28, 29]. Hypoxia can induce ROS production that causes oxidative damage to molecules, cell organelles, and tissues. Many authors speculate that an imbalance between pro- and antioxidants results in an increased level of oxidative degradation of biomolecule products, such as lipid peroxidation products [30]. In our study, different models of hypoxia have been used to evaluate the cellular events associated with hypoxic injury. We induced hypoxia *via* various methods such as asphyctic, haemic (NaNO_2_), and circulatory (NaF) in mice.

In the haemic model, NaNO_2_ induces methemoglobinemia, and in the circulatory model, NaF increases the blood histamine content and decreases the oxygen-carrying capacity and finally inhibits mitochondrial respiration, which demonstrated that more than 90% of Mg^2+^ in the cell is bounded to adenine nucleotides, especially ATP [5]. Chemical hypoxia is distinguished by a reduction of electron carriers in mitochondria and depletion of cell ATP leading to cell ROS formation and, eventually, disruption of the plasma membrane permeability barrier with failure of cell viability [31]. It has been reported that inhibition of ATP-dependent ion transporters after ATP depletion associated with hypoxic conditions results in variation in the levels of cytosolic ions. After the hypoxia onset, a rise in cytosolic free Ca^2+^ occurs, which activates Ca-dependent degradative enzymes that lead to cell death. Also, chemical hypoxia can cause a significant increase in free Na^+^ and H^+^ in the cell [32–34]. It was stated that during hypoxia, oxygen-free radicals are produced, and peroxidation of proteins and membrane lipids contributes to cell damage. Administration of MgSO_4_ to the induced hypoxic mice showed significant attenuation of oxidative stress biomarkers compared to the control groups. Magnesium sulfate is a noncompetitive and voltage-dependent antagonist of the *N*-methyl-D-aspartate (NMDA) receptor-ion channel that may block all pathways and has been revealed to be neuroprotective in patients suffering from acute stroke and/or in various animal models of brain injury. Moreover, it was demonstrated that pretreatment with MgSO_4_ attenuates ROS generation and oxidative damage in the brain cells following induced hypoxia [35]. Our results are compatible with other studies that have shown MgSO_4_ decreases oxidative damage in the brain. The observations of oxidative damage to the membrane simultaneous with mitochondrial dysfunction lend credence to the assumption that an alteration of the mitochondrial membrane is a critical step in hypoxia-induced cell death. It seems that the administration of MgSO_4_ prevents the fall of Mg content in the brain and attenuates the hypoxia-induced neuronal mitochondria membrane damage. These results may appear to be a conflict with previous studies indicating no effect of MgSO_4_ in induced hypoxia on cerebral injury in animals [36, 37].

In the present study, we demonstrated that pretreatment of mice with MgSO_4_ before hypoxia induction not only attenuates the increased oxidative damage in the mouse brain but also improves mitochondria function by different doses of MgSO_4_ which is an essential distinction of our investigation with the previous studies. It was stated that irreversible brain damage in the acute phase of hypoxia could be due to primarily necrotic changes. In regard to hypoxic tissue, diminished cytosolic Mg^2+^ is associated with increased damage and formation of ROS while increases are related to the reverse [35]. The mechanism that Mg exerts its antioxidative effect is unclear, but it may be associated with inhibition of iron-driven lipid peroxidation [38]. Another feasibility is that Mg^2+^ blocks the excessive influx of Ca^2+^ by binding to the NMDA receptor ion channel, known as a trigger of oxygen free radical producing pathways, such as phospholipases, cyclooxygenase, and lipoxygenase [39]. It has been reported that Mg^2+^ alters the neuron sensitivity to an oxidative insult [40].

Our results are in agreement with prior studies that have indicated MgSO_4_ has a beneficial role in the decrease of oxidative stress biomarkers [6, 25, 35, 41]. It has been demonstrated that blockade of the ionic conductance and preventing of calcium ion influx during the NMDA receptor are the mechanism of MgSO_4_ prophylactic action in hypoxia [42]. Administration of MgSO_4_ before or after the exposure to hypoxia prevents the reduction in ATP levels in cerebral tissue [43]. It has been shown that acidosis can be created after hypoxia in cell tissues and consequence of that depletion of mitochondrial energy, induction of proteolytic enzymes, and oxidative damage can occur that finally result in cell death [44]. Therefore, magnesium improves myocardiocyte metabolism and energy, inhibits the accumulation of calcium, and ultimately prevents myocardial cell death, which enhances the delivery of oxygen to the hypoxic tissues, especially the brain, and prevents the cells from hypoxic damage. Moreover, regarding different beneficial results reported for Mg and magnetic Mg (^25^mg) via increase of the creatine kinase activity (2- to 4-fold) and ATP level, it is obvious that Mg by enhancement of both substrate and oxidative phosphorylation pathways can increase the ATP synthesis and prevent the oxidative damage and cell death [41, 45].

It was previously confirmed that following hypoxia, ROS generation, protein, and lipid peroxidation of the neuronal cell membrane and cell membrane dysfunction were increased in the animal's brain [35]. The present changes in LPO, GSH, PC, and mitochondrial function in our study support oxidative damage potential of induced hypoxia by different methods (Table 1 and Figures 4–6). Previous studies have shown that the generation of various free radicals such as nitric oxide, as a consequence of hypoxia, can increase in the cell [46]. Therefore, complex processes such as ATP depletion, the release of excitatory amino acids, the formation of ROS, and alkoxyl radicals are the series of intracellular events due to hypoxic cell injury. Moreover, previous studies have demonstrated that Mg is involved in ATP-dependent pump activity such as Na/K-ATPase and Ca-ATPase, which are sensitive to membrane lipid peroxidation [47, 48]. Therefore, Mg is useful in balancing the ATP and reduction of oxidative stress and has positive effects on cellular hypoxia. Practically, a dose of 600 mg/kg of MgSO_4_ showed the effective potential of antioxidants. Our findings showed that MgSO_4_ at dose 600 mg/kg improves hypoxia-induced oxidative stress status in mitochondria much better than other doses which may be due to more improvement of intracellular Mg levels and mitochondrial ATP.

Finally, this study demonstrated that during hypoxia, neuronal mitochondria oxidative damage can occur associated with a decrease of survival time in mice. Pretreatment with MgSO_4_ attenuated protein and lipid peroxidation and increased mitochondrial function in mice afflicted by different methods of hypoxia. The results of this study support the conclusion that Mg may be of worth in increasing survival time and preventing the mortality associated with asphyxiation.

## 5. Conclusion

MgSO_4_ showed an excellent protective effect against hypoxia in all tested models. Notably, it produced significant and dose-dependent beneficial effects on survival time and oxidative damage in different models of induced hypoxia.

## Figures and Tables

**Table 1:** Evaluation effects of different doses of MgSO_4_ on lipid peroxidation, protein carbonyl formation, and glutathione content against induced hypoxia by different methods.

Parameter ⟶	Group	LPO (*μ*g/mg protein)	PC (*μ*g/mg protein)	GSH (*μ*g/mg protein)
Hypoxia type ↓				
Asphyctic	Con (NS)	0.8770 ± 0.036	1.320 ± 0.097	0.270 ± 0.021
	Phenytoin	0.7560 ± 0.033	0.8527 ± 0.035^∗^	0.1187 ± 0.010^∗^
	Mg 400	0.3000 ± 0.096	0.7573 ± 0.035^∗^	0.1813 ± 0.045
	Mg 500	0.6270 ± 0.027	0.7883 ± 0.028^∗^	0.2123 ± 0.042^∗^
	Mg 600	0.5020 ± 0.210^∗^	0.6873 ± 0.188^∗∗^	0.1233 ± 0.007

Circulatory (NaF)	Con (NS+NaF)	0.3468 ± 0.038	1.532 ± 0.426	0.1174 ± 0.011
	NaF+Mg 400	0.2307 ± 0.026	0.4417 ± 0.032^∗^	0.1077 ± 0.005
	NaF+Mg 500	0.2470 ± 0.062	0.5247 ± 0.074^∗^	0.1143 ± 0.014
	NaF+Mg 600	0.1470 ± 0.010^∗^	0.7007 ± 0.060	0.1163 ± 0.004

Haemic (NaNO_2_)	Con (NS+NaNO_2_)	0.6373 ± 0.039	1.532 ± 0.426	0.148 ± 0.022
	NaNO_2_+Mg 400	0.4913 ± 0.044	0.4343 ± 0.118^∗^	0.0950 ± 0.006
	NaNO_2_+Mg 500	0.2920 ± 0.051^∗^	0.8483 ± 0.192	0.0846 ± 0.004^∗^
	NaNO_2_+Mg 600	0.2860 ± 0.112^∗^	1.081 ± 0.152	0.0993 ± 0.005

Data are presented as mean ± SD of 6 animals in each group. ^∗^*P* < 0.05 and ^∗∗^*P* < 0.01: significantly compared with each control group.

## Data Availability

The current study is the result of a dissertation code 437, School of Pharmacy, Mazandaran University of Medical Sciences, Sari, Iran. The data that support the findings of this study are available from the corresponding author, upon reasonable request.
